# Retrospective evaluation of the clinical performance of direct composite restorations using the snow-plow technique: Up to 4 years follow-up

**DOI:** 10.4317/jced.55639

**Published:** 2019-11-01

**Authors:** Ailreza Borouziniat, Hossain Khaki, Sara Majidinia

**Affiliations:** 1 Dental Research Center, Mashhad University of Medical Sciences, Mashhad, Iran. Department of restorative dentistry, School of Dentistry, Mashhad University of Medical Sciences, Mashhad, Iran; 2 Student Research Committee, School Of Dentistry, Mashhad University Of Medical Sciences, Mashhad, Iran. Post graduate student of orthodontics, School of Dentistry, Mashhad University of Medical Sciences, Mashhad, Iran; 3 Dental Materials Research Center, Mashhad University of Medical Sciences, Mashhad, Iran. Department of restorative dentistry, School of Dentistry, Mashhad University of Medical Sciences, Mashhad, Iran

## Abstract

**Background:**

To evaluate the clinical performance of direct composite restorations using the snowplow technique with up to 4 years of follow-up.

**Material and Methods:**

In this retrospective study, 101 class II composite restorations which were placed in permanent molars (n = 19) and premolars (n = 82) in 85 patients by the snowplow technique, were evaluated according to the modified USPHS criteria. A Kaplan-Meier analysis with a 95% confidence level was used to calculate the survival probability.

**Results:**

Of the 101 teeth examined, tooth failure was identified in 8 teeth due to secondary caries (3%), marginal gap (2%), marginal discoloration (1%) and restoration breakdown (2%) which required replacement of the whole restoration. Sixteen teeth achieved a Bravo score and just needed to be repaired. The results of the Kaplan-Meier analysis regarding overall survival estimates of composite-treated teeth using the snowplow technique at 1-, 2-, 3- and 4- year follow-ups were 99%, 96.2%, 89.6% and 79% respectively. The median survival times of composite restorations using the snowplow technique were 46.49 ± 11.47 month.

**Conclusions:**

This retrospective study showed that application of a flowable composite as a liner using the snowplow technique does not improve the clinical survival of posterior composite restorations.

** Key words:**Snowplow, composite restoration, success rate.

## Introduction

The use of resin composite as a posterior restorative material has markedly increased over the past decade as a result of material improvement. However, polymerization shrinkage and marginal adaptation remain unavoidable problems in composite restorations (1). Polymerization shrinkage of the composite resin can result in breakdown of the adhesive bonds causing the development of a marginal gap especially at the gingival margins of class II restorations because of the minimal or total absence of enamel (2,3). In addition, the viscosity of composite resin plays an important role in the marginal adaptation of composite resin to the cavity walls especially in areas with difficult accessibility. To improve the quality of the restoration margin, the preparation be prepared by filling without voids or porosities. The greatest number of porosities was found in a highly-viscous resin composite. Opdam *et al.* (4) showed that when the resin composites were injected, the quality of the margin improved and the volume of porosities decreased.

In an attempt to reduce polymerization shrinkage, methods such as different curing protocols, use of an incremental technique and application of lining materials have been suggested. Fusayama *et al.* (5) suggested the use of self-cured composites for dentinal gingival margins. Aboushala *et al.* (6) demonstrated that glass ionomer liners reduce marginal microleakage. Olmez *et al.* (7) confirmed the efficiency of flowable liner in improving the marginal adaptation of composite restorations. It has been proposed that flowable composite liners act as a method of relieving the stress associated with polymerization shrinkage. Because of their low filler loading, flowable composites exhibited a lower modulus of elasticity and better stress-buffering capacity than hybrid composite resins that ultimately lead to a better marginal seal of flowable composites (8,9).

Although the reduced viscosity of flowable composite may improve the adaptation to preparation, this may have some adverse effects. The flowable composites exhibit greater polymerization shrinkage due to their lower filler content and this may disrupt the bond to cavity walls (10-12). Frankenberger *et al.* (10) demonstrated that application of flowable composite may increase the incidence of gingival overhangs. In addition, use of a flowable liner has been shown to weaken the strength of the overlying polymerized restorative composite. Furthermore, the radiopacity of many flowable composite materials is not sufficient to detect the presence of voids or recurrent caries under radiographic examination (13).

Recently, Boruziniat *et al.* (14) conducted a meta-analysis and concluded that application of flowable composite couldn’t reduce the microleakage of ‘class II’composite restorations or improve the clinical performance. However in their study the flowable liner was cured before placement of the hybrid composite.

Opdam *et al.* (15) suggested use of the snowplow technique in which most of the flowable composite and therefore its potential disadvantages are removed from the cavity. Instead only a small amount of flowable resin composite remains in the areas of the cavity in which the higher viscosity resin composite does not completely adapt to the preparation and that otherwise may have been voids. Therefore this technique may reduce void formation and increase marginal adaptation.

To the best of our knowledge there have been no clinical studies to date which evaluated this technique, however this study aimed to evaluate the long-term survival of posterior composite resin restorations using the snowplow technique.

## Material and Methods

-Patient selection

For this retrospective study, 85 patients (30 male and 55 female) were selected according to pre-determined inclusion criteria from the total patients registered to a private dental office from 2010 to 2015. These inclusion criteria were as follows: presence of at least one class II composite restoration using the snowplow technique and a follow-up period of at least 12 months. In addition, all details of restorative procedures and the reasons for any failures should be recorded.

-Restorative procedures

All direct class II composite restorations were carried out by a dentist specializing in restorative dentistry (AB) under rubber dam isolation. Cavities were prepared using diamond burs. low-speed steel burs were used to remove carious tissue. Preparations were restricted to carious tissue elimination, no bevels were made. In deep cavities the axial wall was protected with a thin layer of calcium hydroxide (Dycal; Dentsply Siirona, York, PA, USA) and then a thin layer of resin modified glass-ionomer (Fuji II LC, GC Corp., Tokyo, Japan) was applied over it. All cavities were acid etched using 35% phosphoric acid (Ultra etch, Ultradent GmbH, Germany) and the single bond adhesive(3M ESPE, USA) was applied according to the manufacturers’ instructions. Then an initial thin layer of flowable composite (G-anial flow(GC EUROPE, Belgium) or Z350 flow(3M ESPE, USA) ) (approximately 0.25 mm) was placed over the gingival floor of the prepared cavity. This layer was not light cured at this stage, but rather an initial increment of heavily-filled restorative resin was pushed in to unset the flowable resin composite. Most of the flowable resin composite was displaced by the restorative composite and subsequently removed from the prepared cavity and the combined increment of flowable composite and restorative resin composite was photo-activated using an LED curing unit (Bluephase C8, Ivoclar Vivadent, Schaan, Liechtenstein) at 800 mw/cm2 for 40 s. The rest of the cavity was restored incrementally with restorative resin composite (G-anial, GC EUROPE or Z350,3M ESPE); each increment was light cured for 40 s. The restorations were finished using fine-grit diamonds and rubber points with aluminum oxide polishing paste. After finishing and polishing, all surfaces of the restoration were etched for 10 s, rinsed and dried. A hydrophobic resin (Margin bond, Ivoclar Vivadent) was applied and light cured for 20 s.

-Evaluation and statistical analysis

After the protocol was approved by the Committee on Human Rights Related to Human Experimentation, Mashhad University, clinical evaluation of class II restorations was initiated. The patients were invited by telephone calls and email to visit the practice for evaluation. Patients signed a written informed consent prior to the start of the clinical evaluation, and two calibrated researchers (AB & HK) involved in the study carried out the examination. Eighty-five patients (55 female and 30 male, aged 19 to 49 (mean = 29 ± 2) agreed to participate in the study. These patients had 101 posterior composite restorations.

The history of the restorations was initially achieved from the dental records. Some patient-related information was recorded, such as name, gender, date of birth, presence of parafunction, caries risk, treatment date, size of the cavity (MOD or MO/DO), application of pulp protection, tooth type, failure date and reason for failure (if applicable). The restorations were then clinically evaluated using an explorer and dental mirror, according to the United States Public Health Service criteria (USPHS). The surfaces were dried with an air stream before evaluation. In any cases where the evaluators disagreed, they reached an agreement by a new combined evaluation. Most patients in the practice underwent a complete bi-annual periapical radiographic exam, which was assessed by the examiners. Additional radiographs were only made when necessary to complement the clinical evaluation, in order to avoid unnecessary radiation exposure for the patients.

In the current study, failure was defined as loss or fracture of the restoration, severe sensitivity or pulp problems, presence of recurrent caries or any Charlie or Delta scores according to USPHS (United States Public Health Service) criteria.

Statistical analysis was carried out using SPSS for Windows 19.0 statistical package (SPSS Inc., Chicago, IL, USA). A Kaplan-Meier analysis with a 95% confidence level was used to calculate the survival probability.

## Results

Descriptive data of findings are presented in  Table 1. Of the 101 teeth examined in the present study, tooth failure was identified in 8 teeth due to secondary caries (3%), a marginal gap (2%), marginal discoloration (1%) and restoration breakdown (2%) which required the replacement of the whole restoration, while 16 teeth were given a Bravo score and just needed to be repaired.

**Table 1:**
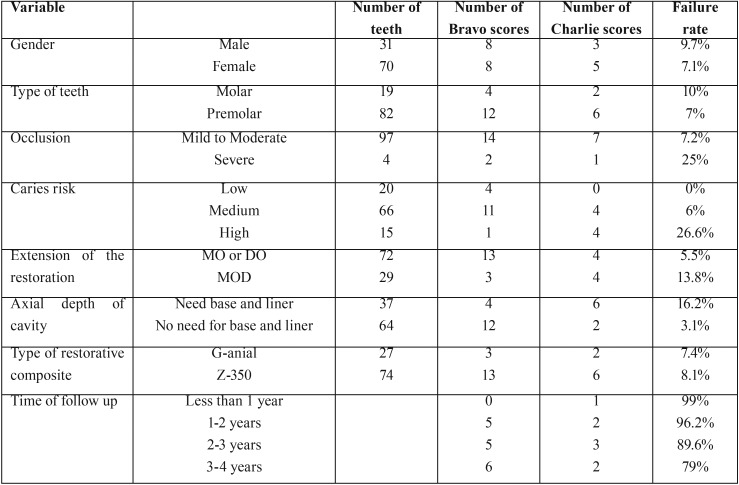
Descriptive data.

The results of the Kaplan-Meier analysis regarding overall survival estimates of composite-treated teeth using the snowplow technique at 1-, 2-, 3- and 4- years were 99%, 96.2%, 89.6% and 79% respectively. The median survival times of composite restorations with the snowplow technique were 46.49 ± 11.47 months.

In the current study, a higher failure rate was observed in molar teeth and in MOD restorations. This study also showed that with the snow plow technique the failure rate in patients at high risk of caries was higher than in those with medium and low risks.

## Discussion

The results of this retrospective study were based on the historical data that was available in the dental records, on patients’ reports and on patient evaluation at the latest follow-up visit for the current condition of the teeth according to USPHS criteria.

Overall survival estimates of posterior composite restorations using the snowplow technique at 1, 2, 3 and 4 years were 99%, 96.2%, 89.6% and 79% respectively and the median survival time was 3.9 years.

Different studies have reported that the median annual failure rate of posterior composite restorations using the conventional technique range from 1 to 3.7% (16,17). However this study showed a 3.9% median annual failure rate for composite restorations using the snowplow technique, which is almost in the same range as the conventional technique. In the snowplow technique the flowable composite was cured together with the following, first increment of hybrid composite. It was speculated that co-curing maximizes the stress-absorbing ability of the flowable composite as the elastic modulus develops concomitantly with the curing of both increments and is, therefore, not already high when curing of the hybrid composite is initiated. Peutzfeldt *et al.* (18) in an *in vitro* study reported a significantly lower amount of microleakage with the snowplow technique compared with the conventional technique.

In this study the main reason for restoration failure was secondary caries, and marginal discoloration and restoration fracture were the second most common, which is in accord with many other studies (19,20).

Several variable factors potentially influenced the survival of posterior composite restoration. Opdam and Rodolfo *et al.* noted that the longevity of restorations directly affected by the tooth type, with restorations in premolars showing better performance than in molars (17,21,22). Opdam *et al.* (17) also reported a higher survival rate for MO/OD preparations than MOD preparations. In the current study, a higher failure rate was observed in molar teeth and in MOD restorations, considering the importance of the amount of remaining tooth structure and the forces applied to the restoration.

It has been shown that Patients’ caries risk significantly influence the survival of the restorations (17,23). Restorations in a high caries-risk patients had a failure rate twice more than low-risk patients(23). This study also showed that with the snow plow technique the failure rate in patients at high risk of caries was higher than in those with medium and low risks. Therefore, although snowplow technique seems to create a better adaptation to the gingival wall and so reduce the secondary caries occurrence, caries risk still seems to play an important role in secondary caries occurrence.

Since fracture of a restoration is another reason for failure, it is, therefore, likely that bruxing habits play a major role in fatigue development in the tooth-restoration complex, which in long term result in fracture.

Although laboratory studies showed significantly different mechanical behavior among different composite materials, in clinical studies with up to 17-year follow-up (24), no significant differences in performance were observed. This finding indicated that clinical performance of different composite materials may vary significantly only when the late failing behavior of composite restorations is taken into consideration. The results of this study are in line with a previous study (24) and showed no difference between two types of restorative composites.

As the operator’s skill significantly influences the survival of a restoration, consequently we suggest that in problematic cases in which access to the entire cavity walls, especially the gingival floor, are difficult (such as endodontic teeth), application of flowable composite as a liner with the snowplow technique can enhance the adaptation of the composite restoration.

One of the limitations of the current study is the unequal follow-up periods. However, the Kaplan-Meier method analysis made it possible to manage this limitation since the survival probability is calculated each time a failure occurs.
